# Metabolomics Profiling of Serum and Urine from Chuanzang Black Pigs with Different Residual Feed Intake

**DOI:** 10.3390/ani14162323

**Published:** 2024-08-12

**Authors:** Xiang Zhou, Chongying Li, Zongze He, Hongwei Liu, Man Wang, Jian He

**Affiliations:** School of Life Science and Engineering, Southwest University of Science and Technology, Mianyang 621010, China; zhouxiang21wb@163.com (X.Z.); 15308116700@163.com (C.L.); xxzongze@gmail.com (Z.H.); lhwqq_71@tom.com (H.L.); 15183525782@139.com (M.W.)

**Keywords:** Chuanzang black pigs, residual feed intake, serum, urine, metabolome

## Abstract

**Simple Summary:**

Heretofore, a series of research has targeted enhancing feed efficiency and identifying dependable feed efficiency biomarkers, yet less research has focused on improving RFI in Chuanzang black pigs. Therefore, our study facilitates a deeper understanding of the differences in serum and urine metabolites of Chuanzang black pigs with different residual feed intakes in seeking to identify differential metabolites with residual feed intake. Our study used a nontargeted metabolomics approach to analyze pig metabolite markers by differences in Chuanzang black pigs’ serum and urine metabolites, providing new insights into the biological differences in residual feed intake selection in Chuanzang black pigs. We believe this paper would be of great interest to breeders of animals.

**Abstract:**

This study was conducted to evaluate associations of blood variables and urine variables with different residual feed intakes (RFIs) in growing Chuanzang black (CB) pigs. A total of 228 growing CB boars from 99 days were used. The same basal diet was offered ad libitum and individual feed intake and body weight were measured over a period of 181 d. The CB pigs were categorized based on their residual feed intake values, with six individuals each from the high and low ends selected and divided into two groups: the low residual feed intake group (LS) and the high residual feed intake group (HS). Serum and urine samples were collected at the end of the experiment for determination of metabolomics profiling. Results showed that there were significantly different metabolites in serum and urine of different RFI groups (fold-change, FC > 2.0 or FC < 0.5, and *p* < 0.05), and 21 metabolites were identified in serum and 61 in urine. Cluster analysis showed that 20 metabolites were up-regulated and one metabolite was down-regulated in serum; 44 metabolites were up-regulated and 17 metabolites were down-regulated in urine. Kyoto Encyclopedia of Genes and Genomes analysis showed that the differential metabolites of serum were enriched in linoleic acid metabolism, and the differential metabolites of urine were enriched in steroid hormone biosynthesis, taurine and hypotaurine metabolism, and primary bile acid biosynthesis. The correlations between serum metabolites and urine metabolites indicated a significant positive correlation between all fatty acyls in serum metabolites and L-glutamate in urine. However, no compelling genetic or blood biomarkers have been found to explain the differences in RFI, suggesting multiple approaches to effective feed use in pigs. This study provides new insights into the subsequent assessment of RFI by metabolomics profiling, as well as the development of novel feed additives for the factors that will facilitate future research directions in CB pigs.

## 1. Introduction

Due to the fact that feed costs form the highest proportion of costs in total farming expenses, cultivating breeds with high feed efficiency has become an effective method for cost reduction and increased productivity. Chuanzang black (CB) pigs, a newly hybridized breed developed in China, have been officially introduced into industrial farming. Indigenous Chinese pig breeds commonly exhibit poor growth performance, low slaughter rates, and low lean meat ratios [1]. In this scenario, hybridization can effectively enhance the growth rate, feed efficiency, and lean meat ratio of indigenous Chinese pig breeds. CB pigs are a novel breed developed in China in 2014 through hybridization with traditional indigenous pig breeds crossing the sow (Tibet × Meishan) with an intermediate Berkshire sire and a Duroc terminal sire, overcoming the shortcomings of Tibetan pigs such as small size, poor appearance, and low reproductive capacity, while aggregating the traits of strong disease resistance and high fertility of Tibetan pigs and imported pigs. Although the feed efficiency of hybrid pigs has been enhanced, it is influenced by individual variations, feeding environment, and feeding methods. Little is known about the metabolic markers between CB pigs with varying feed efficiency in intensive farming conditions.

Improving feed efficiency is the key direction for future high-quality development of the pig industry. Residual feed intake (RFI) as a feed efficiency trait, compared to feed conversion ratio, compensates for the lack of feed intake needed in maintenance and production that cannot be calculated from the feed conversion ratio [2]. RFI was first proposed by Koch et al. [3] as an indicator of the difference between the actual feed intake of animals and that required for maintenance. In the past century, the indicators for measuring feed conversion efficiency of pigs have been continuously optimized, especially with the emergence of electronic feeding equipment, and RFI has been gradually applied to the genetic breeding of pigs. RFI, as a moderately heritable trait with a good selection response, can improve production efficiency through scientific and reasonable continuous selection [4]. In addition to genetic factors, feed efficiency is influenced by factors such as feed processing technology, the digestibility of feed components, feed additives, environment, and body composition [5]. To enhance pig feed efficiency and farming efficiency, exploration of biomarkers for improved feed efficiency is necessary. With the maturation of metabolomics technology, exploring its relationship with economically relevant traits in animals has become a research trend. In animal metabolomics research, the identification of biomarkers for predicting feed efficiency is becoming increasingly common. A study found that biological processes related to lipid metabolism, vitamin D, and glucose metabolism were closely associated with the RFI of DLY (Duroc × Landrace × Yorkshire) pigs [6]. Liaubet et al. [7] found differences in plasma metabolites related to nitrogen, tryptophan metabolism, and energy metabolism in piglets with different residual feed intake (RFI). Myo-inositol and proline were identified as potential markers for predicting different RFI levels, with LRFI piglets having lower concentrations of myo-inositol and higher concentrations of proline, indicating shorter maturation time. Grubbs et al. [8] identified serum proteins as biomarkers that expand the feed efficiency biomarker database by comparing serum markers in fattening pigs with varying RFI. The differences in physical condition, dietary structure, rearing environment, and other factors of the animals may affect the screening of potential metabolic biomarkers. Integrating relevant metabolic data and establishing a related omics biomarker atlas can provide data support for future improvement of feed efficiency with greater accuracy.

A series of research has targeted enhancing feed efficiency (FE) and identifying dependable FE biomarkers, yet less research has focused on improving RFI in CB pigs. Therefore, the objectives of this research were to identify the associations of blood variables and urine variables with different RFIs in CB pigs to provide reliable RFI markers of differential metabolites and improve the breeding production efficiency of pigs.

## 2. Materials and Methods

### 2.1. Animals, Diets, and Experimental Design

All experimental procedures involving the use of animals were approved by the Animal Welfare Committee of Southwest University of Science and Technology (Mianyang, China). This study involved 288 CB pigs at about 40 kg weight with the same weaning batch and similar body conditions from the Wudu boar farm of Sichuan Tieqi Lishi Food Co., Ltd., reared in 16 independent feeding stations within the same building. Each station housed 18 pigs fed ad libitum with a corn/soybean meal/miscellaneous meal diet (Table 1). The basal diet was based on the NRC (2012) and China’s local pig nutrition standard (DB51/T 1971–2015 https://std.sacinfo.org.cn/gnocDb/queryInfo?id=433719A14183776C2381E888A773E90E accessed on 5 August 2024). Uniform feeding management and vaccination procedures were followed without the use of antibiotics during the experiment.

Each feeding station identified pigs using electronic ear tags, recorded feed intake and weight changes from 99 (approximately 43 kg) to 181 days old, and calculated feed conversion ratio and daily weight gain. A multivariate linear regression equation was established based on initial weight (OnBW), average daily weight gain (ADG), and average daily feed intake (ADFI), and then the theoretical feed intake was determined using this regression equation [9]. Residual feed intake was computed as:

RFI = ADFI − (β0 + β1 × OnBW + β2 × ADG)

in which β0 = the intercept, β1 = the partial regression coefficient of ADFI on initial weight, β2 = the partial regression coefficient of ADFI on average daily gain traits. 

On the 181st day of age, the test pigs were categorized based on their residual feed intake values, with 10 individuals each from the high and low ends selected and divided into two groups: the low residual feed intake group (LS) and the high residual feed intake group (HS). The residual feed intake data of these selected individuals are presented in Table 2.

### 2.2. Sample Collections

At the end of the experimental period (d181), blood and urine samples were collected before feeding in the morning. Blood samples were obtained from the anterior vena cava using procoagulation 20 mL tubes, centrifuged at 3500 r/min for about 15 min, and the supernatant was placed into a 1 mL centrifuge tube. Vulval stimulation was used to induce CB pigs to urinate. Urine samples were subsequently stored in 1.5 mL centrifuge tubes. All the samples were followed by cryopreservation in liquid nitrogen.

### 2.3. Analysis of Serum and Urine Metabolites

Metabolome analyses were finished by a commercial company named Novogene (Beijing, China). The samples (100 μL) were placed in the tubes and 80% methanol and 0.1% formic acid were added to the tubes and mixed. Then the sample was centrifuged at 15,000× *g* at 4 °C for 20 min, and then transferred into an Eppendorf tube. Supernatant was diluted to concentration containing 53% methanol using ultrapure water for LC-MS analysis [10,11]. The same volume from each sample was taken for the quality control (QC) samples.

UHPLC-MS/MS analyses were performed using a Vanquish UHPLC system (ThermoFisher, Oberhausen, Germany) coupled with an Orbitrap Q Exactive^TM^ HF mass spectrometer (ThermoFisher, Germany) in Novogene Co., Ltd. (Beijing, China). Samples were injected onto a Hypesil Gold column (ThermoFisher, Oberhausen, Germany) (100 × 2.1 mm, 1.9 μm) using a 17-min linear gradient at a flow rate of 0.2 mL/min. The eluents for the positive polarity mode were eluent A (0.1% FA in water) and eluent B (methanol). The eluents for the negative polarity mode were eluent A (5 mM ammonium acetate, pH 9.0) and eluent B (methanol). The solvent gradient was set as follows: 2% B, 1.5 min; 2–100% B, 12.0 min; 100% B, 14.0 min; 100–2% B, 14.1 min; 2% B, 17 min. The Q Exactive^TM^ HF mass spectrometer (ThermoFisher, Oberhausen, Germany) was operated in positive/negative polarity mode with a spray voltage of 3.2 kV, capillary temperature of 320 °C, sheath gas flow rate of 40 arb, and aux gas flow rate of 10 arb.

Raw data files were processed by filtering, identifying, integrating, correcting, aligning, and normalizing with Compound Discoverer 3.1 (ThermoFisher Scientific, Waltham, MA, USA). The molecular formulas of metabolites were predicted using additive ions, molecular ion peaks, and fragment ion data matrixes, and accurate qualitative and relative quantitative results were obtained using the mzCloud, mzVault, and MassList databases. Statistical analyses were performed using the statistical software R (R version R-3.4.3), Python (Python 2.7.6 version), and CentOS (CentOS release 6.6).

These metabolites were annotated using the KEGG database, HMDB database, and LIPID MAPS database. Partial least squares discriminant analysis (PLS-DA) was performed at metaX. We applied univariate analysis (*t*-test) to calculate the statistical significance. The metabolites with VIP > 1 and *p*-value < 0.05 and fold change (FC) ≥ 2 or FC ≤ 0.5 were considered to be differential metabolites (DFMs). Then, public databases such as MassBank (http://www.massbank.jp/, accessed on 23 July 2022), the Human Metabolome Database (http://www.hmdb.ca/, accessed on 23 July 2022), and METLIN (https://metlin.scripps.edu/, accessed on 23 July 2022) were used to identify metabolites. The functions of these metabolites were studied using the Kyoto Encyclopedia of Genes and Genomes (KEGG) database (http://www.genome.jp/kegg/, accessed on 23 July 2022). Hierarchical clustering analysis and heat map analysis were carried out using the R package v3.4. Due to sample quality and other reasons, we selected six samples in each group for analysis.

### 2.4. Statistical Analyses

Significant differences between the LS and HS groups were analyzed using *t*-tests. *p* < 0.05 was considered significant.

## 3. Results

### 3.1. Serum Metabolome Profling

**Figure 1:**
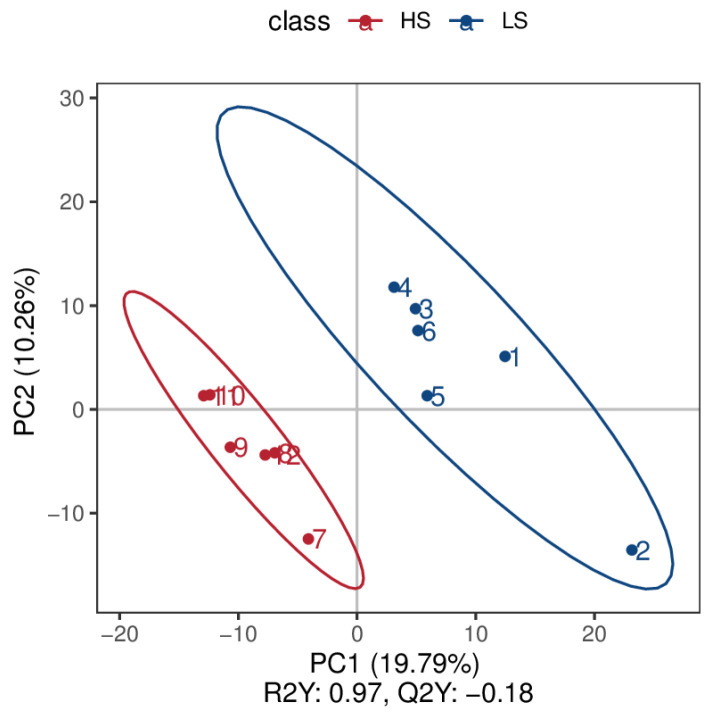
PLS-DA score chart of the HS group (red) and LS group (blue) in serum. LS, the low residual feed intake group; HS, the high residual feed intake group.

**Figure 2:**
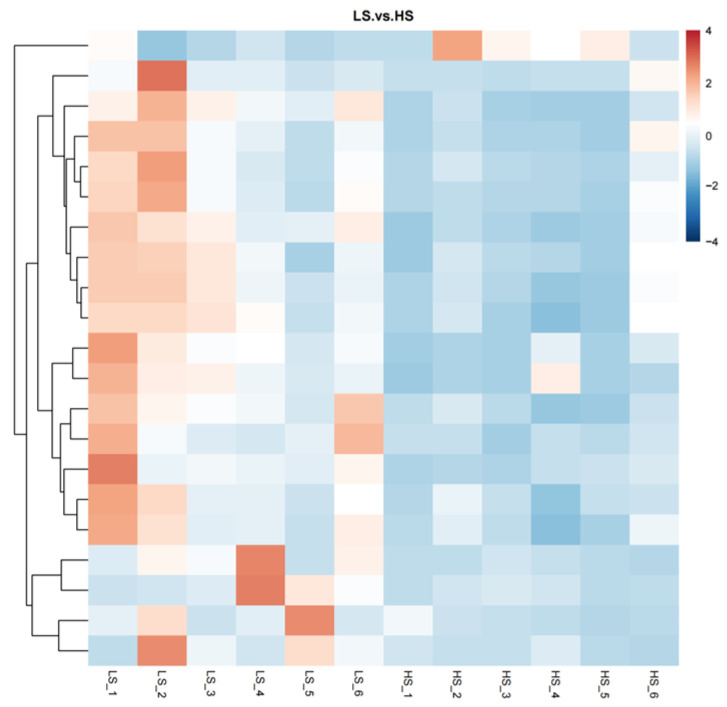
Heatmap of hierarchical clustering analysis for the different metabolites in serum. Each block refers to the abundance of one metabolite from one sample. LS, the low residual feed intake group; HS, the high residual feed intake group.

**Figure 3:**
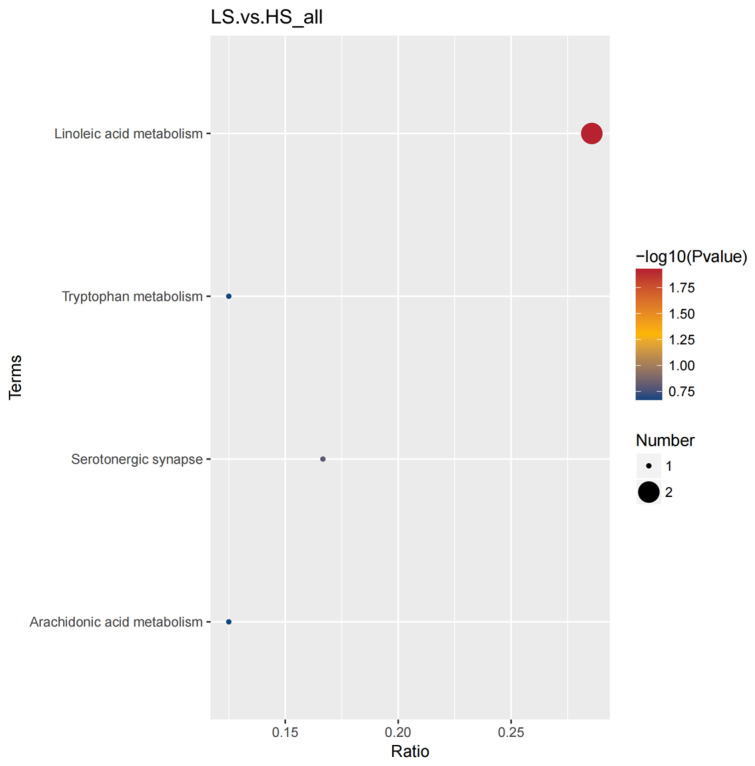
Main differential metabolic pathways in serum. The *x*-axis presents the rich factor. The *y*-axis presents the concentrated KEGG pathway. The size of the bubble indicates the amount of differential abundance metabolites enriched in this pathway, and the color indicates the significance of enrichment.

Through the use of untargeted metabolomics analysis of the serum, a total of 718 metabolites were identified. PLS-DA revealed significant and systematic changes between the LS and HS groups (Figure 1). Differential metabolites were selected based on the criteria of VIP > 1.0, FC > 2.0 or FC < 0.5, and *p*-value < 0.05. In the LS and HS groups, there were 21 significantly different metabolites, with 20 up-regulated and one down-regulated (Table 3). Hierarchical clustering analysis of DFMs was performed with the results shown in Figure 2. The distinction in the red and blue areas between the LS and HS groups was obvious, and the results indicated that there were significant differences in serum metabolites between the LS and HS groups. Found after annotation the metabolite taxonomy, mainly including fatty acyls, glycerophospholipids, organic acids and derivatives, organoheterocyclic compounds, organic oxygen compounds, benzenoids, etc. The KEGG pathway enrichment analysis indicated that the differential metabolic pathway is mainly enriched in linoleic acid metabolism (Figure 3).

### 3.2. Urine Metabolome Profling

**Figure 4:**
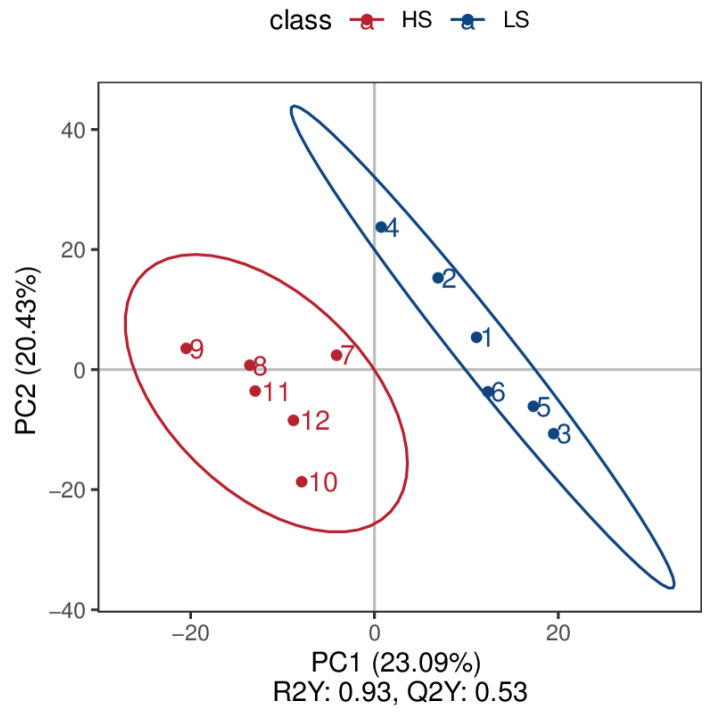
PLS-DA score chart of the HS group (red) and LS group (blue) in urine. LS, the low residual feed intake group; HS, the high residual feed intake group.

**Figure 5:**
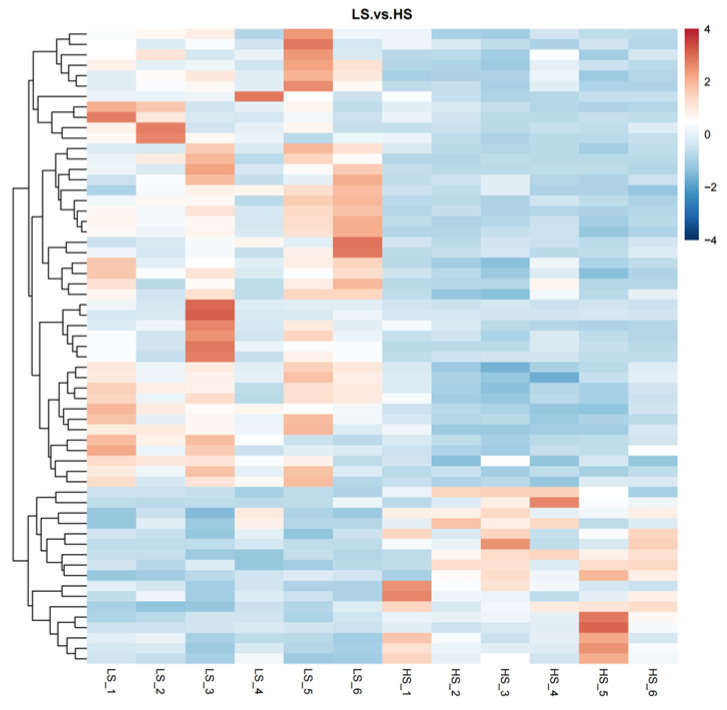
Heatmap of hierarchical clustering analysis for the different metabolites in urine. Each block refers to the abundance of one metabolite from one sample. LS, the low residual feed intake group; HS, the high residual feed intake group.

**Figure 6:**
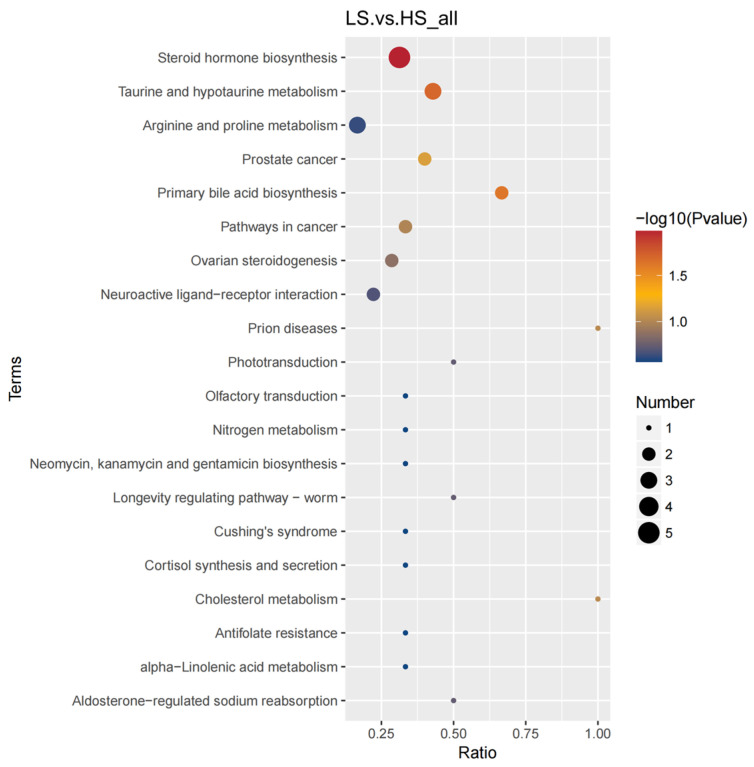
Main differential metabolic pathways in urine. The *x*-axis presents the rich factor. The *y*-axis presents the concentrated KEGG pathway. The size of the bubble indicates the amount of differential abundance metabolites enriched in this pathway, and the color indicates the significance of enrichment.

Through the use of untargeted metabolomics analysis of the urine, A total of 855 metabolites were identified. PLS-DA analysis revealed significant and systematic changes between the LS and HS groups (Figure 4). Differential metabolites were selected based on the criteria of VIP > 1.0, FC > 2.0 or FC < 0.5, and *p*-value < 0.05. In the LS and HS groups, there were 61 significantly different metabolites, with 44 up-regulated and 17 down-regulated (Table 4). Hierarchical clustering analysis of DFMs was performed with the results shown in Figure 5. The distinction in the red and blue areas between the LS and HS groups was obvious, and the results indicated that there were significant differences in urine metabolites between the LS and HS groups. The metabolite taxonomy was found after annotation, mainly including organic acids and derivatives, sterol lipids, fatty acyls, organoheterocyclic compounds, benzenoids, organic oxygen compounds, nucleosides, nucleotides, and analogues, etc. The KEGG pathway enrichment analysis indicated that the differential metabolic pathway is mainly enriched in steroid hormone biosynthesis, taurine and hypotaurine metabolism, and primary bile acid biosynthesis (Figure 6).

### 3.3. Correlations between Serum Metabolites and Urine Metabolites

**Figure 7:**
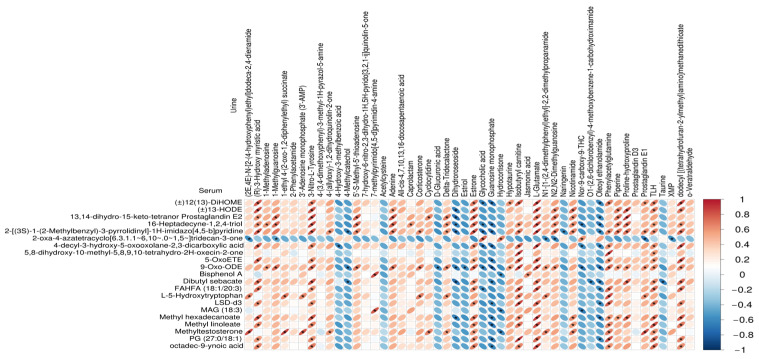
Heat map of correlations between serum differential metabolites and differential metabolites in urine. The redder the color, the stronger the positive correlation, the bluer the color, the stronger the negative correlation, and the flatter the color, the higher the absolute value of the correlation; * indicates *p* < 0.05.

Correlations of the top 50 serum and urinary metabolites were analyzed by Pearson’s correlation coefficient (Figure 7). In serum differential metabolites, methyl linoleate, methyltestosterone, and dibutyl sebacate are positively correlated with L-glutamate (Glu), phenylacetylglutamine, and estrone in urine differential metabolites, and negatively correlated with dihydroroseoside, glycocholic acid, and guanosine monophosphate in urine differential metabolites. The result revealed that the differential metabolites in serum showed a significant correlation with the significant differential metabolites in urine. 

## 4. Discussion

In the present study, the metabolites of the serum mainly including lipids and lipid–like molecules are the major energy source in pig feed, and also affect the feed efficiency of pigs [12]. As a differential metabolic pathway, linoleic acid metabolism is involved in inflammatory response, immune regulation, and cell signaling [13]. Linoleic acid is a polyunsaturated fatty acid (PUFA) that cannot be synthesized in the body, and pigs obtain it primarily from vegetable oils, soybeans, and corn in their feed. Bruininx et al. [14] found that low utilization of fatty acids by fattening pigs increased the energy intake of fattening pigs, which ultimately led to an increase in the energy required to maintain body weight. In our experiment, the upstream metabolites of the linoleic acid metabolism pathway did not differ significantly between the LS and HS groups; however, the downstream 9-Oxo-ODE and (±)12(13)-DiHOME differ significantly between the two groups. 9-Oxo-ODE and (±)12(13)-DiHOME were significantly higher in the LS group than in the HS group, which indicated that the utilization of fatty acids in the LS group was higher than that in the HS group. Therefore, LRFI finishing pigs only need lower energy to maintain body weight. At the same time, it was found that 12(13)-DiHOME, produced by brown fat during exercise, can promote fat metabolism and improve glucose tolerance [15]. 9-Oxo-ODE is a monounsaturated fatty acid metabolite that has been found to reduce gastrointestinal bacterial load [16]. The difference may prove that the pigs in the LS group have better intestinal flora, which can better regulate lipid metabolism. This may explain the difference between the two groups in the linoleic acid metabolic pathway. There are insufficient data to support the relationship of the linoleic acid metabolism pathway and metabolites with residual feed intake, and further research is needed to explore the relationship.

In the urine metabolome analysis, the differential metabolites were mainly concentrated in organic acids and derivatives and lipids and lipid-like molecules. Based on these metabolites, we confirmed three pathways from the KEGG database that were significantly related to RFI, including steroid hormone biosynthesis, taurine and hypotaurine metabolism, and primary bile acid biosynthesis. Lipid metabolism and body composition are important factors influencing RFI [17]. Zhao et al. [18] found that energy metabolism may mediate the biosynthesis of fatty acids and steroid hormone biosynthesis and then affect feed efficiency. Some enzymes in the 17β-hydroxysteroid dehydrogenase family regulate the generation of androgen and estrogens through the interconversion of active and inactive forms of steroids [19]. Estrone and estradiol can inhibit orexin signaling and reduce voluntary feed intake in pigs, while orexin can also act as a signal for estrogen activation, exhibiting a pattern of interaction similar to steroidogenesis [20]. In our experiment, estrone increased in the LS group, and the relevant changes in metabolite content in steroid hormone biosynthesis may be responsible for the different RFIs. The metabolism of taurine and hypotaurine plays an important role in cholesterol metabolism, the nervous system, and cell membrane stability [21,22]. Liu et al. [23] found that taurine can improve the feed efficiency and improve the liver and intestinal health of weaned piglets. Notably, Wang et al. [24] also obtained similar experimental results, which also found that taurine improved the antioxidant capacity of piglets and the microbiota of the cecum. In addition, the downstream product of taurine, Glu, was higher in the LS group than in the HS group. Taurine undergoes metabolization through the action of taurine-2-oxoglutarate transaminase to produce Glu. Glu is one of the most abundant amino acids in animal tissues. Glu can improve the growth or production performance of swine. Meanwhile, Glu promoted the proliferation and differentiation of intestinal stem cells and improved intestinal health through the epidermal growth factor receptor/extracellular regulated protein kinase pathway [25,26]. He et al. [27] found that Glu is the major nutrient for chickens to support their maximum growth and feed efficiency. In addition, earlier studies found that the feed efficiency of finishing pigs was proportional to the Glu content in the diet [28]. A common problem with fattening pigs is that routine diets lead to excessive natural deposition of subcutaneous white adipose tissue. Addition of monosodium glutamate to the diet was found to reduce triglyceride concentrations in plasma and white adipose tissue in growing pigs [29]. Moreover, Hu et al. [30] found that the addition of Glu increased intramuscular fat deposition and had no effect on subcutaneous fat mass. Meanwhile, the correlation analysis indicated a significant positive correlation between Glu and all fatty acyls in serum metabolites. These results indicated that Glu is involved in lipid metabolism, and this result may be achieved by an increased lipolysis and reduced lipogenesis in white adipose tissue, as well as by stimulating the oxidation of fatty acids and glucose in skeletal muscle. Thus, Glu improved the RFI of CB pigs by regulating the lipid metabolism and energy distribution in the body. Primary bile acids are produced by cholesterol in the liver and, after binding to glycocholic acid or taurine, drain into the gallbladder through the bile duct and are further released into the duodenum after feeding [31]. Bile acids can promote the absorption of nutrients in the intestine, especially the lipids and lipid-soluble vitamins in the intestine The vast majority of the bile acids are eventually recovered in the terminal ileum and returned to the liver by the veins [32]. Yan et al. [33] found that after reducing the feeding frequency, the fecal microbiota was affected, which then changed the bile acid profile and ultimately improved the feed efficiency of growing pigs. By analyzing the fecal metabolism of sows with different RFIs, Wu et al. [6] found that the three metabolites associated with bile acid synthesis were significantly negatively associated with RFI traits. Meanwhile, our study also found that glycocholic acid and taurine in the differential metabolic pathway of primary bile acid biosynthesis were significantly reduced in the LRFI group. Although few studies investigated the effect of primary bile acid biosynthesis on feed efficiency, primary bile acid biosynthesis plays an important role in promoting nutrient absorption, energy homeostasis, and metabolic regulation, which deserves further investigation.

Correlation analysis of serum metabolites and urine metabolites indicated a significant positive correlation between all fatty acyls in serum metabolite and Glu in serum. The result may be related to the decrease in residual feed intake prompting increased fat mobilization to meet the energy requirements of CB pigs’ growth activities. This difference in serum metabolic state parameters suggests that CB pigs adopted different metabolic adaptation strategies to meet the need for growth. Meanwhile, fatty acids can improve the cellular metabolism and protect the intestinal mucosa [13]. The difference in RFI caused an improvement in the function of the gut barrier of CB pigs with LRFI, while increasing the function of nutrient metabolism.

## 5. Conclusions

Our study used a nontargeted metabolomics approach to analyze pig metabolite markers by differences in CB pigs’ serum and urine metabolites, providing new insights into the biological differences in RFI selection in CB pigs. This study identified important pathways that are closely associated with RFI, including linoleic acid metabolism, steroid hormone biosynthesis, taurine and hypotaurine metabolism, and primary bile acid biosynthesis. The correlation results indicated that serum differential metabolites of CB pigs with different RFIs were associated with urinary differential metabolites, indicating that these metabolites may mediate the regulation of RFI. However, more studies are needed to understand the molecular mechanisms of RFI of CB pigs, providing new avenues for further research.

## Figures and Tables

**Table 1:** Feed ingredients and nutrient levels of the basal diet.

Ingredient and Nutritive Level	40–75 kg	75–105 kg
Ingredient (%)		
Corn	32.40	37.20
Wheat	10.00	10.00
Crumbs	10.00	12.00
Wheat bran	12.00	7.30
paddy	10.00	10.00
soya bean meal	5.80	3.80
corn DDGS	5.00	5.00
Corn germ meal	8.00	8.00
Alfalfa grass particles	3.00	3.00
Limestone	0.90	1.00
CaHPO_4_	1.00	0.73
NaCl	0.40	0.40
L-Lysine-HCl (70%)	0.60	0.61
DL-Methionine (99%)	0.00	0.01
L-Threonine	0.10	0.11
L-Tryptophan (25%)	0.10	0.14
choline chloride	0.10	0.10
Premix ^1^	0.60	0.60
Total	100.00	100.00
Nutritive levels (%)		
Crude protein	13.5	12.50
Corase fiber	5.01	4.68
Crude ash	5.75	4.96
Digestible energy, MJ/kg	13.27	12.72
Metabolic energy, MJ/kg	12.52	12.44
Calcium	0.70	0.65
Total phosphorus	0.63	0.56
Digestible Lys	0.70	0.66
Digestible Met	0.21	0.20
Digestible Thr	0.43	0.41
Digestible Try	0.13	0.13

^1^ Premix is provided per kilogram of feed: vitamin A, 5500 IU; vitamin VD3, 1000 IU; vitamin E, 50 IU; vitamin K3, 2 mg; vitamin B1, 3.6 mg; vitamin B2, 4.8 mg; vitamin B12, 0.2 mg; niacin, 24 mg; pantothenic acid, 18 mg; biotin, 0.1 mg; folacin, 0.5 mg; Mn, 32 mg; Zn, 80 mg; Cu, 10 mg; I, 1.2 mg; Se, 0.3 mg; Fe, 100 mg.

**Table 2:** Feed efficiency of CB pigs with different surplus feed intake.

Items	LS	HS	SEM	*p*-Value
Initial body weight (kg)	43.385	42.897	0.752	0.76
Final body weight (kg)	106.570	105.770	2.203	0.86
Average daily gain (kg/day)	0.786	0.776	0.023	0.83
Average daily feed intake (kg/day)	2.871	3.203	0.087	0.06
Feed conversion ratio	3.665	4.137	0.071	<0.01
RFI	−0.195	0.180	0.049	<0.01

Note: All traits in this table were analyzed with a pen as the experimental unit and presented as the mean and standard error of the means (SEM) (*n* = 10). Abbreviations: RFI—residual feed intake.

**Table 3:** Identification of significant differential metabolites in serum.

Metabolites	FC ^1^	VIP ^2^	*p*
9-Oxo-ODE	3.93	2.21	<0.01
(±)13-HODE	3.70	2.12	<0.01
2-[(3S)-1-(2-Methylbenzyl)-3-pyrrolidinyl]-1H-imidazo[4,5-b]pyridine	2.14	2.02	<0.01
Methyl hexadecanoate	2.10	1.97	<0.01
16-Heptadecyne-1,2,4-triol	2.74	1.95	<0.01
MAG (18:3)	5.33	2.03	<0.01
13,14-dihydro-15-keto-tetranor Prostaglandin E2	2.25	1.91	0.01
Dibutyl sebacate	2.10	1.88	0.01
(±)12(13)-DiHOME	2.50	1.79	0.01
PG (27:0/18:1)	3.83	1.68	0.01
LSD-d3	2.63	1.67	0.02
FAHFA (18:1/20:3)	2.67	1.65	0.02
4-decyl-3-hydroxy-5-oxooxolane-2,3-dicarboxylic acid	2.59	1.70	0.02
Methyl linoleate	2.21	1.62	0.02
Methyltestosterone	2.76	1.56	0.03
Bisphenol A	3.35	1.98	0.03
octadec-9-ynoic acid	2.30	1.53	0.04
L-5-Hydroxytryptophan	2.41	1.53	0.04
5,8-dihydroxy-10-methyl-5,8,9,10-tetrahydro-2H-oxecin-2-one	2.67	1.48	0.04
5-OxoETE	2.52	1.44	0.05
2-oxa-4-azatetracyclo[6.3.1.1~6,10~.0~1,5~]tridecan-3-one	0.45	1.44	0.05

^1^ FC: fold change; ^2^ VIP: variable importance in the projection.

**Table 4:** Identification of significant differential metabolites in urine.

Metabolites	FC ^1^	VIP ^2^	*p*
1-Methylguanosine	4.95	2.11	<0.01
L-Glutamate	2.19	2.09	<0.01
Nicotinamide	2.93	1.98	<0.01
Adenine	2.62	2.00	<0.01
5′-S-Methyl-5′-thioadenosine	3.37	1.96	<0.01
4-(allyloxy)-1,2-dihydroquinolin-2-one	2.62	1.94	<0.01
N1-[1-(2,4-dimethylphenyl)ethyl]-2,2-dimethylpropanamide	2.61	1.93	<0.01
3′-Adenosine monophosphate (3′-AMP)	3.08	1.89	<0.01
Guanosine monophosphate	0.35	1.97	<0.01
o-Veratraldehyde	10.15	1.89	<0.01
Nor-9-carboxy-δ9-THC	0.28	1.88	<0.01
Proline-hydroxyproline	2.30	1.82	<0.01
XMP	3.01	1.80	<0.01
Estrone	2.34	1.78	<0.01
Prostaglandin E1	2.70	1.83	<0.01
Estriol	0.44	1.83	<0.01
1-Methyladenosine	2.60	1.89	<0.01
Delta-Tridecalactone	3.20	1.82	<0.01
Cyclocytidine	2.18	1.73	<0.01
N2,N2-Dimethylguanosine	2.05	1.80	<0.01
Hydrocortisone	0.48	1.76	0.01
Corticosterone	4.20	1.89	0.01
D-Glucuronic acid	0.40	1.69	0.01
3-Nitro-L-Tyrosine	3.01	1.76	0.01
Piperine	2.69	1.66	0.01
4-Methylcatechol	0.35	1.63	0.01
Prostaglandin D3	4.50	1.69	0.01
Naringenin	0.33	1.56	0.02
2-Phenylacetamide	2.30	1.63	0.02
Jasmonic acid	2.75	1.60	0.02
Dihydroroseoside	0.44	1.65	0.02
1-ethyl 4-(2-oxo-1,2-diphenylethyl) succinate	2.56	1.55	0.02
7-hydroxy-6-nitro-2,3-dihydro-1H,5H-pyrido[3,2,1-ij]quinolin-5-one	2.20	1.52	0.02
Hypotaurine	2.29	1.65	0.02
Glycocholic acid	0.35	1.61	0.02
(2E,4E)-N-[2-(4-hydroxyphenyl)ethyl]dodeca-2,4-dienamide	2.52	1.54	0.02
Oleoyl ethanolamide	0.49	1.52	0.02
Caprolactam	2.99	1.59	0.02
Phenylacetylglutamine	3.32	1.58	0.02
Acetylcysteine	0.41	1.51	0.02
Taurine	0.38	1.52	0.02
TLH	3.51	1.53	0.02
dodecyl [(tetrahydrofuran-2-ylmethyl)amino]methanedithioate	2.46	1.53	0.02
7-methylpyrimido[4,5-d]pyrimidin-4-amine	2.03	1.52	0.03
Isobutyryl carnitine	2.17	1.54	0.03
(R)-3-Hydroxy myristic acid	2.06	1.46	0.03
4-(3,4-dimethoxyphenyl)-3-methyl-1H-pyrazol-5-amine	11.06	1.57	0.03
O1-(2,6-dichlorobenzyl)-4-methoxybenzene-1-carbohydroximamide	0.10	1.52	0.03
4-Hydroxy-3-methylbenzoic acid	0.30	1.49	0.03
All-cis-4,7,10,13,16-docosapentaenoic acid	2.64	1.46	0.03
Artesunate	2.87	1.46	0.03
Hydroxyproline	3.92	1.43	0.03
2-[(carboxymethyl)(methyl)amino]-5-methoxybenzoic acid	2.04	1.50	0.03
cis-4-Hydroxy-D-proline	2.08	1.46	0.03
Kanosamine	2.40	1.52	0.04
Dehydroepiandrosterone	2.48	1.43	0.04
N-Acetyl-L-aspartic acid	2.33	1.50	0.04
Catechol	0.49	1.39	0.04
N1-[3,5-di(trifluoromethyl)phenyl]-2-cyclopentyl-2-phenylacetamide	0.18	1.49	0.04
N-(9-oxodecyl)acetamide	0.44	1.36	0.04
Deisopropylatrazine	2.12	1.35	0.05

^1^ FC: fold change; ^2^ VIP: variable importance in the projection.

## Data Availability

The data used to support the findings of this study can be made available by the corresponding authors upon request.
